# Accurate Analysis of Target Characteristic in Bistatic SAR Images: A Dihedral Corner Reflectors Case

**DOI:** 10.3390/s18010024

**Published:** 2017-12-22

**Authors:** Dongyang Ao, Yuanhao Li, Cheng Hu, Weiming Tian

**Affiliations:** 1 Radar Research Lab, School of Information and Electronics, Beijing Institute of Technology, Beijing 100081, China; 20091295@bit.edu.cn (D.A.); cchchb@bit.edu.cn (C.H.); tianweiming@bit.edu.cn (W.T.); 2 Key Laboratory of Electronic and Information Technology in Satellite Navigation (Beijing Institute of Technology), Ministry of Education, Beijing 100081, China

**Keywords:** dihedral corner reflector, bistatic radar sensor, scattering characteristic, SAR image

## Abstract

The dihedral corner reflectors are the basic geometric structure of many targets and are the main contributions of radar cross section (RCS) in the synthetic aperture radar (SAR) images. In stealth technologies, the elaborate design of the dihedral corners with different opening angles is a useful approach to reduce the high RCS generated by multiple reflections. As bistatic synthetic aperture sensors have flexible geometric configurations and are sensitive to the dihedral corners with different opening angles, they specially fit for the stealth target detections. In this paper, the scattering characteristic of dihedral corner reflectors is accurately analyzed in bistatic synthetic aperture images. The variation of RCS with the changing opening angle is formulated and the method to design a proper bistatic radar for maximizing the detection capability is provided. Both the results of the theoretical analysis and the experiments show the bistatic SAR could detect the dihedral corners, under a certain bistatic angle which is related to the geometry of target structures.

## 1. Introduction

With the rapid development of synthetic aperture radar (SAR) imaging technology, there are a variety of SAR systems [1], including the monostatic SAR systems and the multi-static SAR systems. The most typical system of the multi-static SAR systems is the bistatic SAR, which has been consistently studied for many years [2]. The bistatic SAR system operates with distinct transmitting and receiving antennas implemented on separate platforms [3]. With its flexible configuration, the bistatic SAR can capture more comprehensive scattering properties of the scene targets. Compared with the traditional monostatic SAR system, the bistatic SAR system has unparalleled advantages. For example, the “quiet” operation of the receiver brings good concealment, high security, and strong anti-interference ability. The bistatic SAR system also can detect scattering information from different aspects, which will help target data fusion. Based on these advantages, bistatic SAR system is often used for military and scientific missions [4]. As early as the mid-1990s, the first bistatic SAR experiment was completed by the United States, using the spaceflight and air receivers [5]. In 2002, several bistatic experimental systems using ground stationary, airborne, and spaceborne receivers were developed [6,7], and related imaging algorithms were proposed [8,9]. In 2010, DLR had launched the TanDEM-X SAR satellite forming the bistatic SAR configuration with the previously launched TerraSAR-X, which opened a new era in the spaceborne radar sensor systems [10,11]. In 2013, Beijing Institute of Technology had applied a global navigation satellite system (GNSS) and the ground-based receivers to accomplish a bistatic SAR system, which demonstrates the flexible geometry of bistatic system can obtain more information for the target scene [12]. In the meantime, the imaging algorithm of bistatic SAR is quite different than the traditional monostatic SAR and many research works paid attention to the focusing algorithm. In the recent ten years, many investigations have been published and make the bistatic SAR system become a popular topic in the radar sensor field [13,14,15].

Before the appearance of the bistatic radar system, it is difficult to detect concealed target with low radar cross section (RCS), because of the development of stealth technique. There are many ways to reduce the targets’ RCS in the stealth technique, such as by material and geometry designs [16]. For example, F22 is a flight equipped with many high-tech stealth techniques. One of the most important features is that its shape is strange, because the shape, consisting of unusual settled panels, is designed to avoid forming right-angle structures which can generate high RCS and make it easy to expose in the monostatic radar system [17]. The right-angle structures are often called dihedral corners whose effects are quite common in monostatic radar sensors. There are lots of dihedral corners generated by artificial targets, such as the joints between different flat surfaces [18]. Therefore, the dihedral corners are important structures for radar target detection. Usually, they are composed of two plates which are usually orthogonal. In monostatic radar sensors, the orthogonal dihedral corner has the dihedral effect that generates the main contribution of high RCS. To eliminate the dihedral effect, changing the geometric shape is a basic method and is commonly used in designing stealth targets [19]. Conversely, how to detect stealth targets is an important task in the radar sensor design. To this point, the bistatic radar system becomes a good solution. The anti-stealth ability of bistatic radars has been discussed in [20,21]. However, little attention has been focused on how to design a proper bistatic SAR system and the study of the characteristics of the dihedral corners in the bistatic SAR images. Therefore, a principle for designing bistatic radars and accurate explanations of dihedral corners’ RCS are needed. Although the preliminary idea and the simulations of the conception has been discussed in [22], more detailed theoretical analyses and case validations about the discussion of the relationship between the bistatic SAR and the opening angle, the method for designing a proper bistatic radar, and the validation by real bistatic SAR data are still missing.

In this paper, we consider the dihedral corner reflectors as the entry point, apply the geometrical optics theory to analyze the differences of the radar wave propagation in monostatic and bistatic radar systems, and explain why some low RCS targets in the monostatic radar increase their RCS in the bistatic mode. Firstly, we give a principle of how to design the bistatic angle to make the dihedral corners have higher RCSs. The wave path of the microwave is calculated according to the specular reflection geometry. Secondly, for the quantitative analysis, the RCS formula of dihedral corners is proposed. It mathematically demonstrates the RCS changes with the variation of the bistatic radar geometry. Finally, the data generated by the full-wave method is used to simulate SAR images under different radar systems. The simulation results reveal the behaviors of different dihedral corners in SAR images, which is accordant with the proposed theory and proofs the advantage of bistatic radar sensors. In addition, we have applied TanDEM-X data to verify our theory.

This paper is organized as follows: Section 2 depicts three different types of dihedral corners, which are classified by the opening angle. Then, the wave propagations of these targets are analyzed based on the geometrical optical method. Moreover, the bistatic geometry that can detect high RCS of dihedral angles is designed. In Section 3, the RCS formulas of the dihedral corners are deduced, which is an analytical scattering model based on the parameters of the dihedral corners. The calculated results are compared with the full-wave methods. In Section 4, the experiments in SAR images are conducted, and each type of the dihedral corners is simulated under different radar configurations. The practical data experiments also have been conducted using TanDEM-X data. Section 5 discuss the theory and the experimental results. Finally, the conclusions are drawn in Section 6.

## 2. Dihedral Corner Reflectors with Different Opening Angle

Dihedral structure is a common structure in the remote sensing site. Many targets can be simplified as the combination of dihedral corners. In this paper, the method for analyzing them are mainly based on geometrical optical methods which pursuits the propagation of waves. If no multiple reflection, there is no difference between monostatic radar and bistatic radar. However, when considering the effect of multiple reflection, the characteristics of dihedral corner reflectors in bistatic radar and monostatic radar become different.

Based on the opening angle, the dihedral corners can be divided into three groups: the right angle, the acute angle and the obtuse angle. In the following, the three kinds of reflectors are discussed respectively.

### 2.1. Orthogonal Dihedral Corners

**Figure 1:**
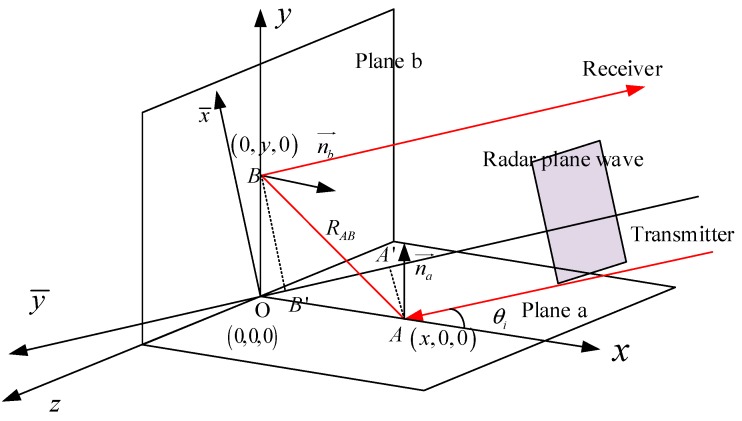
Geometric optical path of the orthogonal dihedral corner.

Orthogonal dihedral corners are widely distributed in scenes, such as the vertical wall and its standing ground, the vertical tree and the ground, the ship and the ocean surface. The orthogonal dihedral corners have strong RCS responses to the monostatic radar. To realize its working mechanism, the geometrical optics method is applied to trace the wave propagation path inside the dihedral corners. The geometric optical path of an orthogonal dihedral corner is shown in Figure 1.

The coordinate system in Figure 1 is set as follows: the vertex of the dihedral corner as the origin, and two planes, a and b, are placed in the *x-z* plane and the *y-z* plane, respectively. When a radar wave illuminates into the corner reflector with incident angle $\theta_{i}$, which is the angle between incident wave and the *x* axis, giving the first illuminated point A in plane a with position (x,0,0), the radar wave will be reflected to point B (0,y,0) in plane b with normal vector $\vec{n_{b}}$ and return with the same angle. The propagation of the radar wave follows the law of specular reflection. In order to analyze the dihedrals’ behavior in SAR images, the distance of the propagation from point A and point B is a vital parameter, which determines the position of targets in SAR images.

To make the computation easier, we set a new coordinate. The new coordinate $\bar{x}\bar{y}z$ sets the line parallel to the radar incident wave as $\bar{y}$-axis. It is equivalent to rotating the local coordinate system xyz by rotating $\pi /2+\theta_{i}$ in the counter clockwise direction. We call it the radar coordinate system. At first, we define the opening angle of the dihedral corner as:

(1) $$2\beta \;=\;\pi -\arccos\left(\vec{n_{a}}\cdot \vec{n_{b}}\right)$$

The modules of vectors in point A and B have the following relationship with the opening angle and the incident angle:

(2) $$\frac{\left|\vec{OA}\right|}{\sin\left(\pi -2\beta -\theta_{i}\right)}=\frac{\left|\vec{OB}\right|}{\sin\theta_{i}}$$

For the orthogonal dihedral corners, $2\beta =\frac{\pi }{2}$. In SAR imaging, the points with the same value in $\bar{y}$ will be considered as a same target [23]. Therefore, only the projection distance on the $\bar{y}$ axis affects the final results. We have:

(3) $$\left|\vec{{OA}^{\prime }}\right|=\left|\vec{OA}\right|\cos\theta_{i}$$

and

(4) $$\left|\vec{{OB}^{\prime }}\right|=\left|\vec{OB}\right|\sin\theta_{i}$$

When the radar wave propagates inside the dihedral, there will be an additional path for the bouncing between point A and point B. The distance is

(5) $$\left|\vec{AB}\right|=\frac{\left|\vec{OB}\right|}{\sin\theta_{i}}\sin\left(2\beta \right)$$

Thus, the phase caused by propagation is

(6) $$\begin{array}{l} \phi =\frac{2\pi }{\lambda }\left(R_{0}-\left|\vec{{OA}^{\prime }}\right|+R_{0}-\left|\vec{{OB}^{\prime }}\right|+\left|\vec{AB}\right|\right)\\ =\frac{2\pi }{\lambda }\left(2R_{0}-\left|\vec{OA}\right|\left(\frac{\cos^{2}\theta_{i}}{\cos\theta_{i}}+\frac{\sin^{2}\theta_{i}}{\cos\theta_{i}}-\frac{1}{\cos\theta_{i}}\right)\right)=\frac{4\pi R_{0}}{\lambda } \end{array}$$

where λ is the wave length of the radar sensor and $R_{0}$ is the distance between radar sensors and the original point.

In (6), all the information in the range direction of the reflector is compressed in the original point. As a result, the corner position produces a very strong echo signal. The strong echo at corner point is the main reason that the orthogonal dihedral corner in the SAR image appears as a high RCS target, which is called as the dihedral effect. The phenomena are common and have been deeply investigated [24]. However, for the dihedrals with weird structures, there are few researches analyzing their scattering characteristics. Next, we will study the obtuse and acute dihedrals that often appears in stealth structures.

### 2.2. Obtuse Dihedral Corners

**Figure 2:**
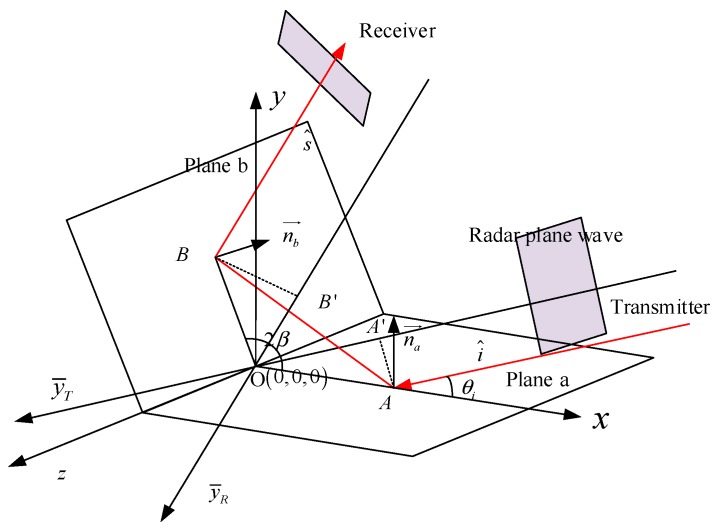
Propagation path of radar wave in the obtuse dihedral corner.

In a monostatic radar case, the orthogonal dihedral corners have high RCS value, which becomes a drawback for target stealth. Therefore, many stealth targets are in non-right angle dihedral structures. In this section, the obtuse dihedral corners are analysed. The propagation path of an obtuse dihedral corner is shown in Figure 2.

Obviously, it is hard for the monostatic radar to receive the echo reflected from the upper plane. To catch this echo, the receiver can no longer be located at the same position with the transmitter. Assuming the opening angle of the dihedral corner is 2β and the incident angle is $\theta_{i}$, the main energy of the radar wave is reflected along the direction of $4\beta +\theta_{i}-\pi$ according to the specular reflection law. Therefore, if we detect the dihedral structure using a radar with a bistatic angle of

(7) α = 4β−π

the strong energy could be obtained, and the RCS will become higher.

When the radar wave hits at point A with incident angle $\theta_{i}$, the location of point A in the transmitter coordinate system is

(8) $$\left|\vec{{OA}^{\prime }}\right|=\left|\vec{OA}\right|\cos\theta_{i}$$

Reflecting from point A, the hitting point in plane b is point B, whose location in the receiver coordinate system is

(9) $$\left|\vec{{OB}^{\prime }}\right|=\left|\vec{OB}\right|\cos\left(\pi -\theta_{i}-2\beta \right)$$

where y is the distance from point 2 to the original point. Based on the law of sines, we have

(10) $$\frac{\left|\vec{OA}\right|}{\sin\left(\pi -\theta_{i}-2\beta \right)}=\frac{\left|\vec{OB}\right|}{\sin\left(\theta_{i}\right)}=\frac{\left|\vec{AB}\right|}{\sin\left(2\beta \right)}$$

Thus, the phase of the echo can be expressed by

(11) $$\begin{array}{l} \phi =\frac{2\pi }{\lambda }\left(R_{0}-\left|\vec{{OA}^{\prime }}\right|+R_{0}-\left|\vec{{OB}^{\prime }}\right|+\left|\vec{AB}\right|\right)\\ =\frac{2\pi }{\lambda }\left(2R_{0}-\left|\vec{OA}\right|\left(\frac{\cos\theta_{i}\cos\left(\theta_{i}+2\beta -\frac{\pi }{2}\right)}{\cos\left(\theta_{i}+2\beta -\frac{\pi }{2}\right)}+\frac{\sin\theta_{i}\sin\left(\theta_{i}+2\beta -\frac{\pi }{2}\right)}{\cos\left(\theta_{i}+2\beta -\frac{\pi }{2}\right)}-\frac{\sin2\beta }{\sin\left(\frac{\pi }{2}+\theta_{i}-2\beta \right)}\right)\right)\\ =\frac{2\pi }{\lambda }\left(2R_{0}-\left|\vec{OA}\right|\left(\frac{\cos\left(\theta_{i}+2\beta -\frac{\pi }{2}-\theta \right)}{\cos\left(\theta_{i}+2\beta -\frac{\pi }{2}\right)}-\frac{\sin2\beta }{\cos\left(\theta_{i}+2\beta -\frac{\pi }{2}\right)}\right)\right)=\frac{4\pi R_{0}}{\lambda } \end{array}$$

In (11), we can see that the obtuse dihedral corner displays a strong point in the vertex in the SAR image under a specific bistatic angle α = 4β−π. If the bistatic angle is not accurate matched, the strong echo cannot be caught.

### 2.3. Acute Dihedral Corners

According to the classification of the opening angle, the acute dihedral corner is discussed in this section and is focused on the multiple reflection, comparing to the obtuse and orthogonal dihedral reflectors where only the double-bouncing occurs.

#### 2.3.1. Double-Bouncing

**Figure 3:**
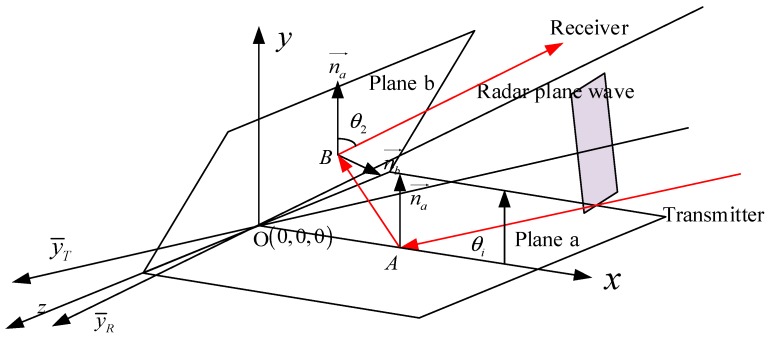
Double-bouncing reflection path of an acute dihedral corner case.

In the case of the double-bouncing, the RCS behavior of the acute dihedral corners has no difference with the other twos. The double-bouncing reflection path of an acute dihedral corner is shown in Figure 3.

**Figure 4:**
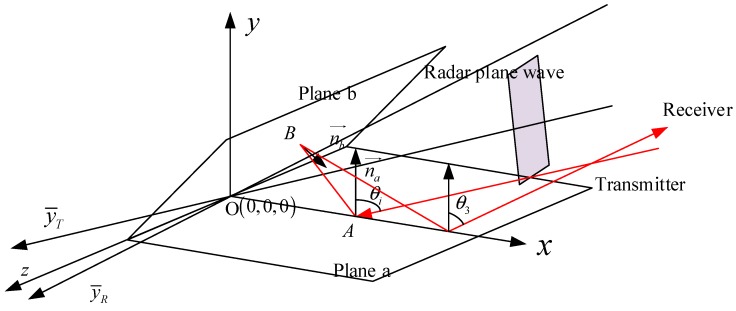
Three-bouncing reflection path of an acute dihedral corner case.

In Figure 3, the emergent angle points to the positive direction of *y* axis. When the opening angle 2β becomes smaller, the red-line echo will not be received by the receiver and it will hit plane a again. Given the angle of the second reflected echo $\theta_{2}=\pi -4\beta +\theta_{i}$, when $\theta_{2}>\pi /2$, the third reflection will appear (Figure 4), and the emergent angle will be $\theta_{3}=4\beta -\theta_{i}$. Continuously, in the case of $\theta_{3}>\pi /2-2\beta$, the forth one occurs.

#### 2.3.2. Multiple-Bouncing

**Figure 5:**
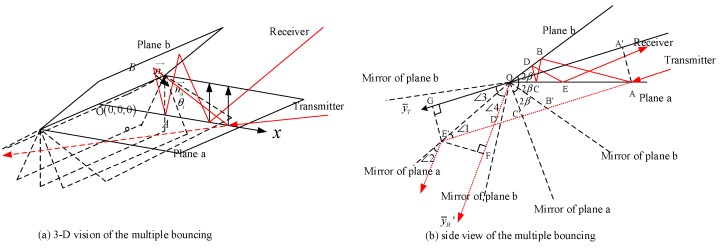
Equivalent propagation of multiple reflection (**a**) 3-D vision of the multiple bouncing; (**b**) side view of the multiple bouncing.

In order to discover the principle of multiple-bouncing in the acute dihedral, the mirror projection of the propagation path is used to analyse the relationship between the incident angle and the times of reflection. The illumination is shown in Figure 5.

In the analysis of multiple bouncing, we use a trick that regarding every specular reflection as a straight light propagation in the mirror domain. Every time the radar wave reflects, we make a mirror of the hitting plane according to the opening angle in the way as shown in Figure 5. Due to the complexity of 3-D vision, the side view is provided as a simple demonstration in Figure 5b. Based on the geometry, we can calculate the wave path distance from incident point A to the emergent point E. No matter how many bounces it occurs, we have the basic geometric relationship, which is

(12) $$\left|\vec{{AE}^{\prime }}\right|=\left|\vec{A^{\prime }G}\right|$$

If we get the strong echo using the receiver located in the direction of ${{\bar{y}}_{R}}^{\prime }$, it is easy to prove that

(13) ΔOAD≅ΔOED

Thus, we have

(14) $$\left|\vec{OG}\right|=\left|\vec{OF}\right|$$

The phase of the multiple bouncing echo can be written as

(15) $$\begin{array}{l} \phi =\frac{2\pi }{\lambda }\left(R_{0}-\left|\vec{{OA}^{\prime }}\right|+R_{0}-\left|\vec{OF}\right|+\left|\vec{{AE}^{\prime }}\right|\right)\\ =\frac{2\pi }{\lambda }\left(2R_{0}-\left|\vec{{OA}^{\prime }}\right|-\left|\vec{OG}\right|+\left|\vec{{AE}^{\prime }}\right|\right)=\frac{4\pi R_{0}}{\lambda } \end{array}$$

Similarly, the final position of the acute dihedral can also be the original point in the bistatic SAR images.

### 2.4. Emergent Angles and Bouncing Times

**Figure 6:**
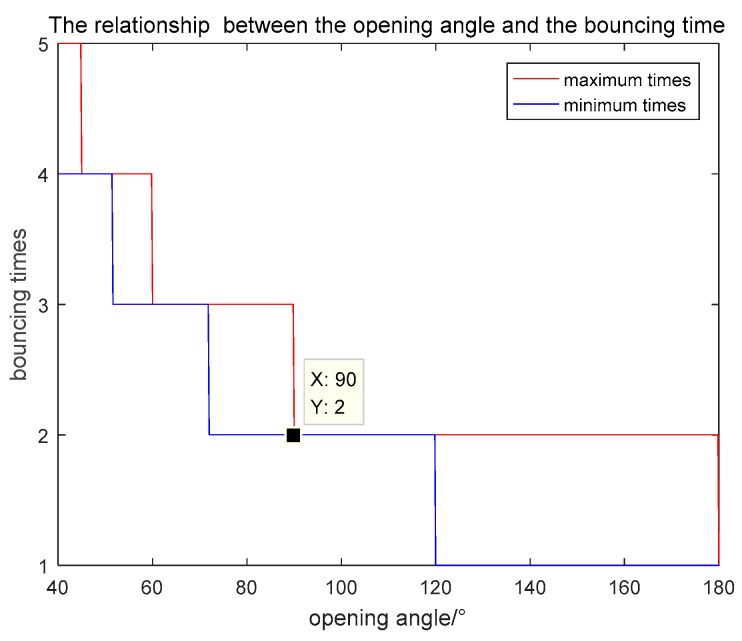
Relationship between the opening angle and the bouncing time.

Due to the existence of the multiple reflection, the bouncing time is a special problem for acute dihedral corners. Using the straight light geometry in Figure 5b and setting the incident angle $0<\theta_{i}<\beta$. When $\beta <\theta_{i}<2\beta$, it is symmetrical where plane b can be regarded as plane a and the coordination can be rotated. Therefore, we only concentrate the incident angle having $0<\theta_{i}<\beta$. If the final emergent wave goes through *n times* bounce, it must obey that

(16) $$2\beta \cdot n>\pi -\theta_{i}>2\beta \cdot \left(n-1\right)$$

and

(17) $$n=\left\lceil \frac{\pi -\theta_{i}}{2\beta }\right\rceil$$

where ⌈ ⌉ stands for rounding up to an integer. Thus, the maximum time of the bounce is $n_{\max}=\left\lceil \frac{\pi }{2\beta }\right\rceil$ when $\theta_{i}=0$, The minimum time of the bounce is $n_{\min}=\left\lceil \frac{\pi -\beta }{2\beta }\right\rceil$ when $\theta_{i}=\beta$. The relationship between the opening angle and the bouncing time is shown in Figure 6.

In Figure 6, we can see that the orthogonal and obtuse dihedral corners have the maximum bouncing time 2. It is consistent with the previous conclusions. For the acute dihedrals, the smaller the opening angle is, the more bouncing time they have. There is a special phenomenon that when the opening angle is in the range from 90° to 120°, the maximum and minimum bouncing time are the same. Whatever the incident angle is, the double bounces always exist in these dihedral corners. For the obtuse dihedrals with the opening angle larger than 120°, different incident angles have different bouncing times. According to (16) and the domain of definition, we can get the relationship between the bouncing time and the incident angle, which is:

(18) $$\max\left(0,\pi -2\beta n\right)<\theta_{i}<\min\left(\beta ,\pi -2\beta \left(n-1\right)\right)$$

Hereto, we can see how the incident angle affect the bouncing time. However, the emergent angle is still unknown.

Based on the straight light propagation in Figure 5b, the emergent angle is formulated as:

(19) $$\theta_{s}=\left\{ \begin{array}{l} \pi -\left(\theta_{i}+2\beta \left(n-1\right)\right),n=2k-1\\ 2\beta n-\pi +\theta_{i},n=2k \end{array}\right.,k\in N^{+}$$

The emergent angle in (19) has two types of presentation which is classified by the bouncing time being even or singular. Having (19), the relationship between the incident angle and the emergent angle is

(20) $$\left\{ \begin{array}{l} \theta_{s}+\theta_{i}=\pi -2\beta \left(n-1\right),n=2k-1\\ \theta_{s}-\theta_{i}=2\beta n-\pi ,n=2k \end{array}\right.,k\in N^{+}$$

Equation (20) gives the criterion for designing the bistatic radar configuration. If the bouncing time is singular, the transmitter and the receiver should fly in the opposite direction to keep the sum of the two angles unchanged. If the bouncing time is even, the transmitter and the receiver should fly in the same direction to maintain the difference of the two angles. Thus, we give the criterion for designing bistatic radars to obtain high RCS of all kinds of dihedrals.

### 2.5. Summary

From the above analysis, all kinds of types of dihedral corners have high RCS values in a specific bistatic radar observation. The strongest pixel in the SAR image locates at the connection of two panels. The analysis is mainly based on the principle of radar wave propagation along the straight line, which are quite concise, but it only gives qualitative conclusions. Thus, the calculation of the dihedral corners’ RCS is also needed to investigate. In the next section, we propose the RCS calculation formula for dihedral corners under bistatic configurations and give simulation and practical experiment data verifications.

## 3. Bistatic RCS Formula of Dihedral Corner Reflectors

**Figure 7:**
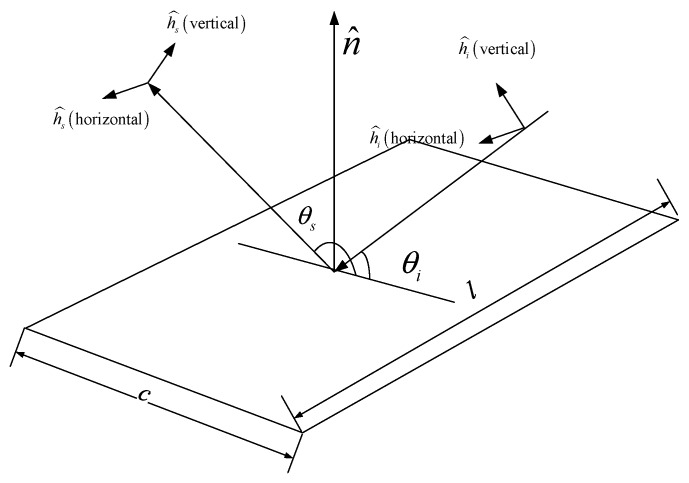
Sketch of the illusion of radar on a plate.

The former section focuses on the propagation path in some specified conditions. It has a general conclusion, but does not give the specific value of the RCS which is a kind of quantitative parameter. Thus, this section uses the quantitative tool to analyse it. To simplify the problem, the edge diffraction of the electromagnetic wave is not considered. The dihedral corner is a structure consisting of two plates. Therefore, at first, we have to analyse the RCS formula of a plate which is shown in Figure 7.

In Figure 7, we assume that the radar beam emitted by the transmitter enters the plate along the unit vector $\hat{i}$ where the polarization direction is ${\hat{h}}_{i}$, the receiver receives the echo along the unit vector $\hat{s}$ where the polarization direction is ${\hat{h}}_{s}$, the normal unit vector of the plate is $\hat{n}$, the incident and reflected angles are $\theta_{i},\theta_{s}$, respectively. Then, according to the physical optical approximation formula mentioned in [18], we have

(21) $$S=\frac{ik^{2}lc}{2\pi }\left(\hat{s}\times {\hat{h}}_{s}\right)\cdot \left(\hat{n}\times {\hat{h}}_{i}\right)e^{ik{\bar{r}}_{o}\cdot \left(\hat{i}-\hat{s}\right)}\sin c\left(\frac{kc\left(\cos\theta_{i}+\cos\theta_{s}\right)}{2}\right)$$

where k=2π/λ, λ is the wave length, ${\bar{r}}_{o}$ is the position vector of the plate centroid, c is the length of the plate and l is the length of the plane.

### 3.1. Theory Analysis of a Dihedral Structure

When the maximum bouncing time is two, the common case for orthogonal and obtuse dihedral corners, the RCS of a dihedral corner composes of four parts: $S_{a},S_{b}$ and $S_{ab},S_{ba}$. $S_{a}$ and $S_{b}$ are the contributions of two planes, $S_{ab}$ and $S_{ba}$ are the contributions of the second bounces for each plane. Therefore, the RCS can be written as the summation of four parts

(22) $$\sigma =\frac{\lambda^{2}}{\pi }{\left|S_{a}+S_{b}+S_{ab}+S_{ba}\right|}^{2}$$

Using (21), we can calculate every component in (22) one by one. At first, the first bounce components for the two single planes can be written as

(23) $$S_{a}=-ika\left(\frac{l}{\lambda }\right)\sin\theta_{i}e^{-ik\frac{a}{2}\left(\cos\theta_{i}+\cos\theta_{s}\right)}\sin c\left[ka\frac{\left(\cos\theta_{i}+\cos\theta_{s}\right)}{2}\right]$$

(24) $$S_{b}=-ikb\left(\frac{l}{\lambda }\right)\sin\left(2\beta -\theta_{i}\right)e^{-ik\frac{b}{2}\left(\cos\left(2\beta -\theta_{i}\right)+\cos g\left(2\beta -\theta_{s}\right)\right)}\sin c\left[kb\frac{\cos\left(2\beta -\theta_{i}\right)+\cos\left(2\beta -\theta_{s}\right)}{2}\right]$$

where a is the length for the plane a and b is the length of plane b.

For the double bouncing components of two planes, they can be regarded as a changed plane in the mirror domain where the multiple reflection is considered to be the straight line propagation. Using the mirror domain method, the RCS components generated by the double bouncing is easy to calculate and the key point is to find the equivalent plane length for both plane a and plane b.

#### 3.1.1. Double-Bouncing

**Figure 8:**
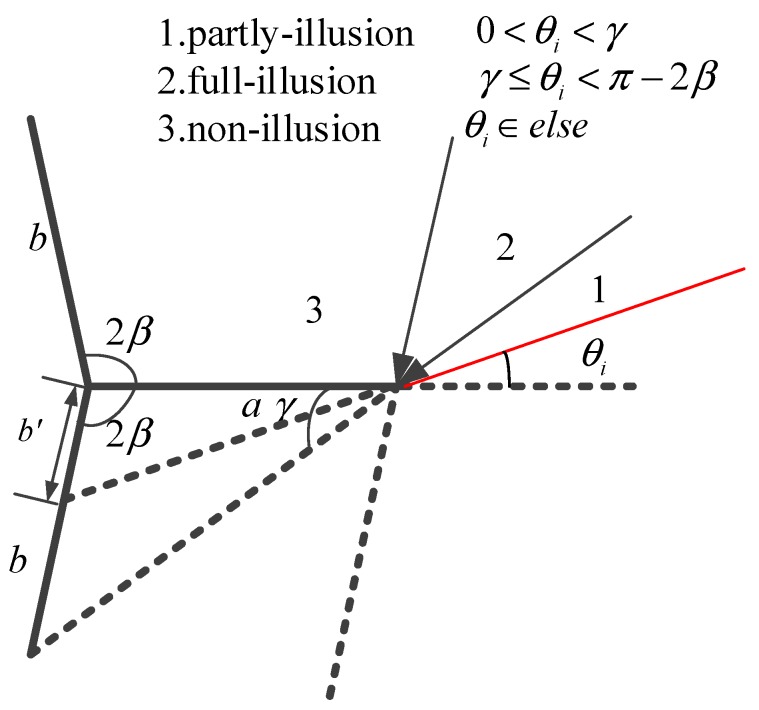
Geometry map for calculating the equivalent length of plane b.

The component $S_{ab}$, which corresponds to the RCS generated by the radar wave hitting in plane a and then reflecting to plane b, is mainly based on the equivalent length of plane b. Figure 8 demonstrates the geometry for calculating the equivalent length and the angles.

In Figure 8, the equivalent length is calculated as follows:

(25) $$b^{\prime }=\left\{ \begin{array}{l} a\frac{\sin\left(\theta_{i}\right)}{\sin\left(2\beta +\theta_{i}\right)},\;0<\theta_{i}<\gamma \\ b,\;\;\gamma \leq \theta_{i}<\pi -2\beta \\ 0,\;\;\;else \end{array}\right.$$

where $\gamma =\arctan\frac{b\sin2\beta }{a-b\cos2\beta }$. Substituting the equivalent length into (21), the value of the secondary scattering from plane b is

(26) $$S_{ab}=-ikb^{\prime }\frac{l}{\lambda }\sin\left(2\beta +\theta_{i}\right)e^{-ikb^{\prime }\left(\frac{\cos\left(2\beta +\theta_{i}\right)+\cos\left(2\beta -\theta_{s}\right)}{2}\right)}\sin c\left[kb^{\prime }\frac{\cos\left(2\beta +\theta_{i}\right)+\cos\left(2\beta -\theta_{s}\right)}{2}\right]$$

**Figure 9:**
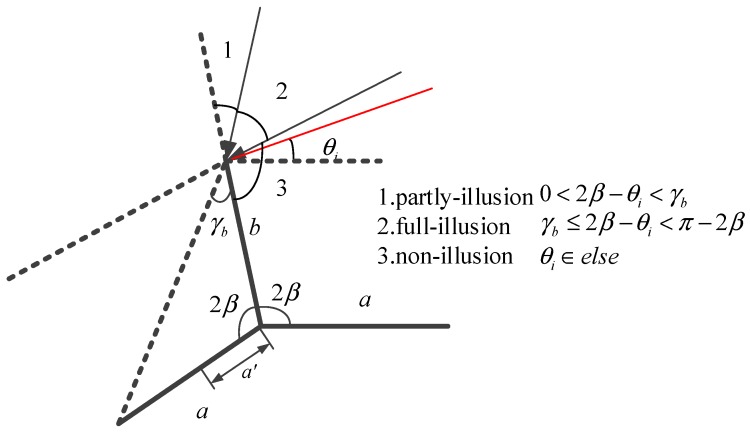
Geometry map for calculating the equivalent length of plane a.

Similarly, the equivalent length of plane a is depicted in Figure 9.

Based on the geometry in Figure 9, the equivalent length of plane a is

(27) $$a^{\prime }=\left\{ \begin{array}{l} b\frac{\sin\left(2\beta -\theta_{i}\right)}{\sin\left(4\beta -\theta_{i}\right)},\;0\leq 2\beta -\theta_{i}<\gamma_{b}\\ a,\;\;\;\gamma_{b}\leq 2\beta -\theta_{i}<\pi -2\beta \\ 0,\;\;\;\;else \end{array}\right.$$

where $\gamma_{b}=\arctan\frac{a\sin2\beta }{b-a\cos2\beta }$. Thus, the secondary scattering value for plane a is

(28) $$S_{ba}=-ika^{\prime }\frac{l}{\lambda }\sin\left(4\beta -\theta_{i}\right)e^{-ika^{\prime }\frac{\cos\left(4\beta -\theta_{i}\right)+\cos\theta_{s}}{2}}\sin c\left[ka^{\prime }\frac{\cos\left(4\beta -\theta_{i}\right)+\cos\theta_{s}}{2}\right]$$

Finally, the RCS formula for the dihedral corners under bistatic radar illumination can be modelled as follows:

(29) $$\sigma =\frac{l^{2}}{4\pi }{\left|\sum_{m=1}^{4}\frac{R_{m}\sin P_{m}\left(e^{-i2Q_{m}}-1\right)}{Q_{m}}\right|}^{2}$$

where $R_{m},P_{m},Q_{m}$ is given in Table 1.

In Table 1, we have obtained the RCS formula of the dihedral angle under bistatic observations. However, it just considers the influence of the double bounce.

#### 3.1.2. Multiple-Bouncing

**Figure 10:**
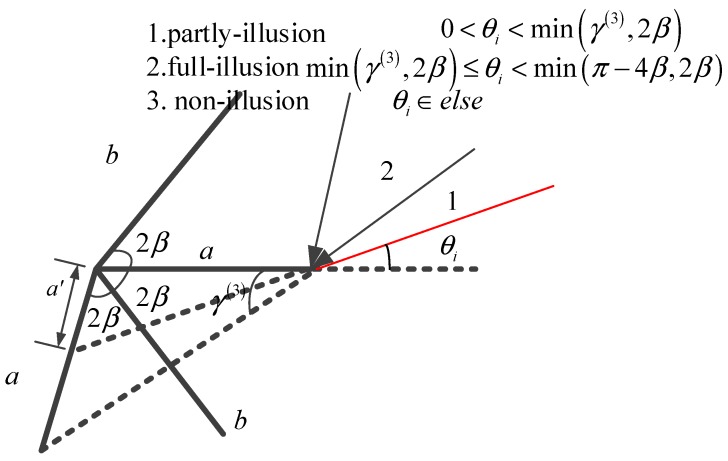
Geometry map of the third reflection.

In the situation of acute dihedrals, the double bouncing is not enough to describe the reflections of the radar wave. The multiple bouncing contributions should be added in the RCS formula. Taking the third reflection as an example, the sketch map is shown in Figure 10.

Expend the formulas for the secondary scattering, the third scattering components for both planes are given as:

(30) $$\begin{array}{l} S_{3a}=-ika^{\left(3\right)}\frac{l}{\lambda }\sin\left(2\beta \cdot 2+\theta_{i}\right)e^{-ika^{\left(3\right)}\left(\frac{\cos\left(2\beta \cdot 2+\theta_{i}\right)+\cos\left(2\beta -\theta_{s}\right)}{2}\right)}\sin c\left[ka^{\left(3\right)}\frac{\cos\left(2\beta 2+\theta_{i}\right)+\cos\theta_{s}}{2}\right]\\ S_{3b}=-ikb^{\left(3\right)}\frac{l}{\lambda }\sin\left(2\beta \cdot 3-\theta_{i}\right)e^{-ikb^{\left(3\right)}\left(\frac{\cos\left(2\beta \cdot 3-\theta_{i}\right)+\cos\left(2\beta -\theta_{s}\right)}{2}\right)}\sin c\left[kb^{\left(3\right)}\frac{\cos\left(2\beta \cdot 3-\theta_{i}\right)+\cos\left(2\beta -\theta_{s}\right)}{2}\right] \end{array}$$

where

(31) $$\begin{array}{l} a^{\left(3\right)}=\left\{ \begin{array}{l} a\frac{\sin\theta_{i}}{\sin\left(4\beta +\theta_{i}\right)},0<\theta_{i}<\min\left(2\beta ,\gamma_{a}^{\left(3\right)}\right)\\ a,\min\left(2\beta ,\gamma_{a}^{\left(3\right)}\right)<\theta_{i}<\min\left(2\beta ,\pi -4\beta \right)\\ 0,else \end{array}\right.,\;\mathrm{with}\;\gamma_{a}^{\left(3\right)}=\arctan\frac{a\sin\left(4\beta \right)}{a-a\cos\left(4\beta \right)}\\ b^{\left(3\right)}=\left\{ \begin{array}{l} b\frac{\sin\left(2\beta -\theta_{i}\right)}{\sin\left(6\beta -\theta_{i}\right)},0<2\beta -\theta_{i}<\min\left(2\beta ,\gamma_{b}^{\left(3\right)}\right)\\ b,\min\left(2\beta ,\gamma_{b}^{\left(3\right)}\right)<2\beta -\theta_{i}<\min\left(2\beta ,\pi -4\beta \right)\\ 0,else \end{array}\right.\;\mathrm{with}\;\gamma_{b}^{\left(3\right)}=\arctan\frac{b\sin\left(4\beta \right)}{b-b\cos\left(4\beta \right)} \end{array}$$

Having the third reflection scattering component and the former discussion in Section 2 and Section 3, we give the final expression for the RCS of dihedral corners as follows

(32) $$\sigma =\frac{l^{2}}{4\pi }{\left|\sum_{n=1}^{N}\left(S_{a}^{n}+S_{b}^{n}\right)\right|}^{2},N=\left\lceil \frac{\pi }{2\beta }\right\rceil$$

where N is the maximum number of bouncing times and

(33) $$S_{a}^{n}=\left\{ \begin{array}{l} -ika^{\left(n\right)}\frac{l}{\lambda }\sin\left(2\beta \cdot \left(n-1\right)+\theta_{i}\right)e^{-ika^{\left(n\right)}\left(\frac{\cos\left(2\beta \cdot \left(n-1\right)+\theta_{i}\right)+\cos\theta_{s}}{2}\right)}\sin c\left[ka^{\left(n\right)}\frac{\cos\left(2\beta \cdot \left(n-1\right)+\theta_{i}\right)+\cos\theta_{s}}{2}\right],n=2k-1\\ -ika^{\left(n\right)}\frac{l}{\lambda }\sin\left(2\beta \cdot n-\theta_{i}\right)e^{-ika^{\left(n\right)}\left(\frac{\cos\left(2\beta \cdot n-\theta_{i}\right)+\cos\theta_{s}}{2}\right)}\sin c\left[ka^{\left(n\right)}\frac{\cos\left(2\beta \cdot n-\theta_{i}\right)+\cos\theta_{s}}{2}\right],n=2k \end{array}\right.,k\in N^{+}$$

where

(34) $$\begin{array}{l} a^{\left(n\right)}=\left\{ \begin{array}{l} a\frac{\sin\theta_{i}}{\sin\left(2\beta \left(n-1\right)+\theta_{i}\right)},0<\theta_{i}<\min\left(2\beta ,\gamma_{a}^{\left(n\right)}\right)\\ a,\min\left(2\beta ,\gamma_{a}^{\left(n\right)}\right)<\theta_{i}<\min\left(2\beta ,\pi -2\beta \left(n-1\right)\right)\\ 0,else \end{array}\right.,\;\mathrm{with}\;\gamma_{a}^{\left(n\right)}=\arctan\frac{a\sin\left(2\beta \left(n-1\right)\right)}{a-a\cos\left(2\beta \left(n-1\right)\right)},n=2k-1\\ a^{\left(n\right)}=\left\{ \begin{array}{l} b\frac{\sin\left(2\beta -\theta_{i}\right)}{\sin\left(2\beta n-\theta_{i}\right)},0<2\beta -\theta_{i}<\min\left(2\beta ,\gamma_{a}^{\left(n\right)}\right)\\ a,\min\left(2\beta ,\gamma_{a}^{\left(n\right)}\right)<2\beta -\theta_{i}<\min\left(2\beta ,\pi -2\beta \left(n-1\right)\right)\\ 0,else \end{array}\right.,\;\mathrm{with}\;\gamma_{a}^{\left(n\right)}=\arctan\frac{a\sin\left(2\beta \left(n-1\right)\right)}{b-a\cos\left(2\beta \left(n-1\right)\right)},n=2k \end{array}$$

The component of the *n*th reflection from plane b is

(35) $$S_{b}^{n}=\left\{ \begin{array}{l} -ikb^{\left(n\right)}\frac{l}{\lambda }\sin\left(2\beta \cdot n-\theta_{i}\right)e^{-ika^{\left(n\right)}\left(\frac{\cos\left(2\beta \cdot n-\theta_{i}\right)+\cos\left(2\beta -\theta_{s}\right)}{2}\right)}\sin c\left[kb^{\left(n\right)}\frac{\cos\left(2\beta \cdot n-\theta_{i}\right)+\cos\left(2\beta -\theta_{s}\right)}{2}\right],n=2k-1\\ -ikb^{\left(n\right)}\frac{l}{\lambda }\sin\left(2\beta \cdot \left(n-1\right)+\theta_{i}\right)e^{-ika^{\left(n\right)}\left(\frac{\cos\left(2\beta \cdot \left(n-1\right)+\theta_{i}\right)+\cos\left(2\beta -\theta_{s}\right)}{2}\right)}\sin c\left[kb^{\left(n\right)}\frac{\cos\left(2\beta \cdot \left(n-1\right)+\theta_{i}\right)+\cos\left(2\beta -\theta_{s}\right)}{2}\right],n=2k \end{array}\right.,k\in N^{+}$$

where

(36) $$\begin{array}{l} b^{\left(n\right)}=\left\{ \begin{array}{l} b\frac{\sin\left(2\beta -\theta_{i}\right)}{\sin\left(2\beta n-\theta_{i}\right)},0<2\beta -\theta_{i}<\min\left(2\beta ,\gamma^{\left(i\right)}\right)\\ b,\min\left(2\beta ,\gamma^{\left(i\right)}\right)<2\beta -\theta_{i}<\min\left(2\beta ,\pi -2\beta \left(n-1\right)\right)\\ 0,else \end{array}\right.,\;\mathrm{with}\;\gamma_{b}^{\left(n\right)}=\arctan\frac{b\sin\left(2\beta \left(n-1\right)\right)}{b-b\cos\left(2\beta \left(n-1\right)\right)},n=2k-1\\ b^{\left(n\right)}=\left\{ \begin{array}{l} a\frac{\sin\theta_{i}}{\sin\left(2\beta \left(n-1\right)+\theta_{i}\right)},0<\theta_{i}<\min\left(2\beta ,\gamma_{b}^{\left(n\right)}\right)\\ b,\min\left(2\beta ,\gamma_{b}^{\left(n\right)}\right)<\theta_{i}<\min\left(2\beta ,\pi -2\beta \left(n-1\right)\right)\\ 0,else \end{array}\right.,\;\mathrm{with}\;\gamma_{b}^{\left(n\right)}=\arctan\frac{b\sin\left(2\beta \left(n-1\right)\right)}{a-b\cos\left(2\beta \left(n-1\right)\right)},n=2k \end{array}$$

In the above analysis, the formulas are divided into two forms due to the parity of the number of the reflection times. For each reflection, they have different local coordinates. Thus, the formulas have this form and it can be proved by the mathematical induction.

**Figure 11:**
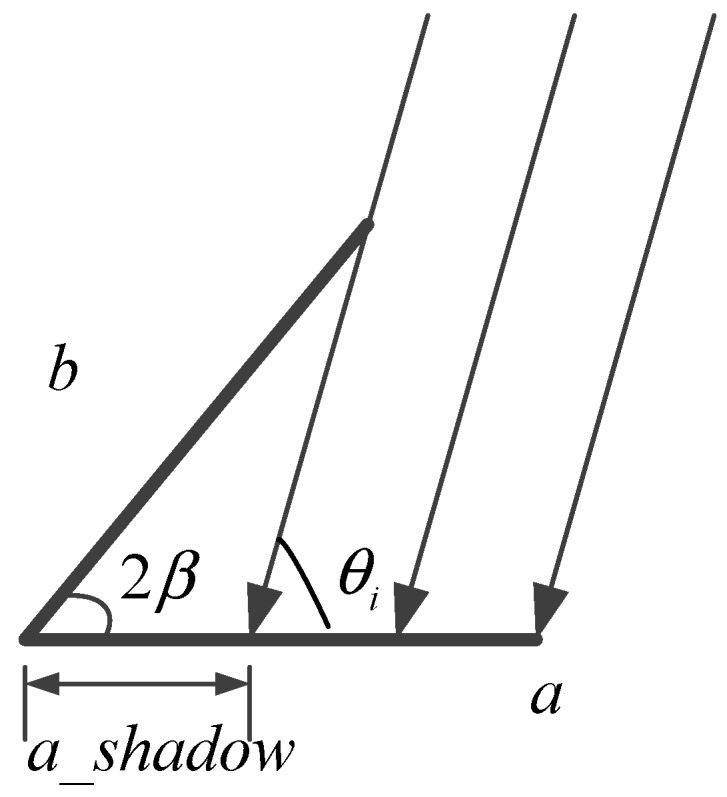
Shadow effect of plane a.

However, we just focus on the incident angle inside the opening angle. For the angles larger than the opening angle, the shadow effect of the dihedrals can influence the RCS. The shadow effect is that plane a blocks the illumination on plane b and vice versa. The shadow effect of plane a is shown in Figure 11.

In Figure 11, the shadow generated by plane b on plane a will not contribute to the final RCS. The length of shadow a is

(37) $$a_{shadow}=\frac{b\sin\left(\theta_{i}-2\beta \right)}{\sin\left(\theta_{i}\right)}$$

For the shadow on plane b, its length can be calculated as

(38) $$b_{shadow}=\frac{-\sin\theta_{i}}{\sin\left(2\beta -\theta_{i}\right)}$$

To remove the shadow influence, we should minus the RCS caused by the shadows. The following formula accomplishes this operation

(39) $$\sigma =\frac{l^{2}}{4\pi }{\left|\sum_{n=1}^{N}\left(S_{a}^{n}+S_{b}^{n}-S_{a_{shadow}}^{n}-S_{b_{shadow}}^{n}\right)\right|}^{2},N=\left\lceil \frac{\pi }{2\beta }\right\rceil$$

where $S_{a_{shadow}}^{n}$ is the result when setting $a=a_{shadow}$ and the same with $S_{b_{shadow}}^{n}$. Hereto, we have obtained the RCS formula for all kinds of dihedral corners.

### 3.2. Verification of the RCS Formula

In order to verify the correctness of these formulas, we use (39) to simulate and test whether it conforms to the conclusion in Section 2, and finally verify whether it is consistent with the result obtained by the Method of Moment (MoM) which is a full wave analysis method [25].

#### 3.2.1. Orthogonal Dihedral Corners

**Figure 12:**
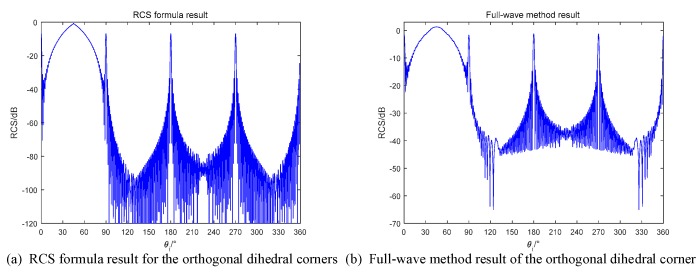
Monostatic RCS of the orthogonal dihedral (**a**) RCS formula in this paper; (**b**) Full-wave method.

For orthogonal dihedral corners, we predict that there will be a strong RCS in the range of 0–90° when using monostatic radars, and in the condition of bistatic radars, the RCS will be reduced in the same angle range. The RCS formula simulation results are compared in Figure 12.

In Figure 12, the shapes of two methods are nearly the same. For the angles in the range of [0,90°], the RCSs in these locate have the components from the secondary scattering. Thus, the peak in this range has larger value than the value in 0°, 90°, 180° and 270°, where the monostatic radar receives the specular reflection of each single plane.

**Figure 13:**
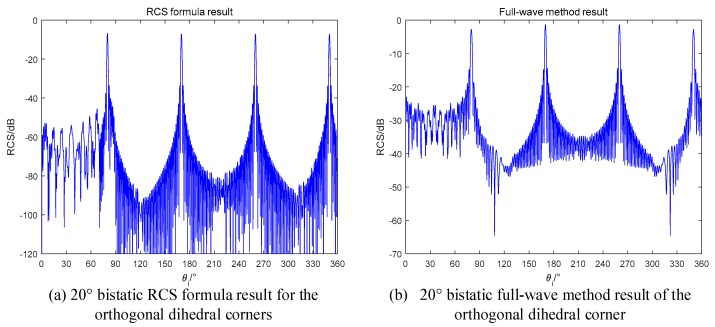
20° bistatic RCS of the orthogonal dihedral (**a**) RCS formula in this paper; (**b**) Full-wave method.

If we use the bistatic radar to detect the orthogonal dihedral, the RCS will be reduced according to the theory in Section 2. To investigate this case, the simulation results are shown in Figure 13. The bistatic radar is with 20° bistatic angle and the transmitter and the receiver are flying in the same direction.

In Figure 13, the range of [0,90°] no longer maintains high values, which is same as predicted. Besides, the RCS formula results are also well fitted with the full-wave method results.

#### 3.2.2. Obtuse Dihedral Corners

**Figure 14:**
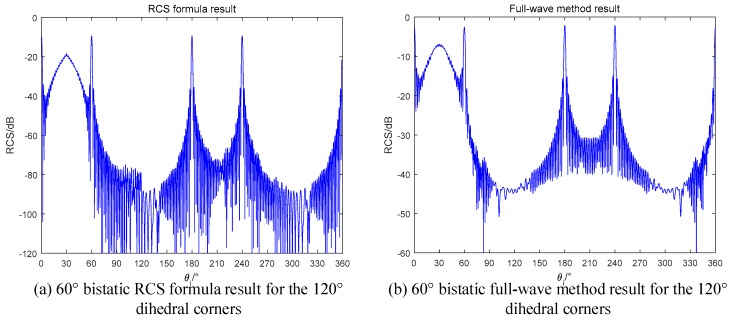
60° bistatic RCS of the 120° dihedral (**a**) RCS formula in this paper; (**b**) Full-wave method.

For obtuse dihedral corners, the maximum scattering times is 2. Based on the conclusions in Section 2, if setting the bistatic angle as $\alpha =\theta_{s}-\theta_{i}=4\beta -\pi$, we can get the RCS curve, such as the result in [0,90°] of monostatic radar for the orthogonal dihedral. Taking a 120° dihedral corner as an example, we use 60° bistatic angle to illuminate it and the results are in Figure 14.

**Figure 15:**
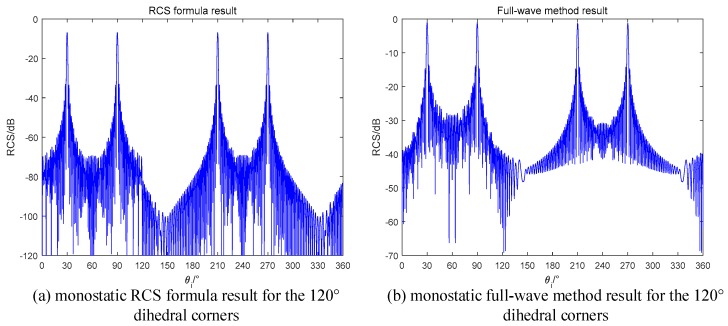
Monostatic RCS of the 120° dihedral (**a**) RCS formula in this paper; (**b**) Full-wave method.

As we predicted, the RCS curve appears a sinc-like envelope in the range of [0,60°] in both the formula method and the full-wave method. The results proof that our radar design is correct. If we use the monostatic radar to observe the obtuse dihedral, the result will be the curves in Figure 15.

In Figure 15, the peak RCS only occurs in the angles which correspond to the single specular reflection for each single plane. The monostatic radar cannot detect the dihedral effect of obtuse dihedrals, which is generated by the mutual scattering of two planes. On the contrary, the obtuse dihedral structures can be used for designing stealth targets for the monostatic radars.

#### 3.2.3. Acute Dihedral Corners

The situation for acute dihedral corners is quite complicate. The bouncing times of can be odd or even, and each case has its own local coordinate. That make the RCS formula has a series of components. To make it easy, we chose the 50° dihedral corner as an example to test our formula. For a 50° acute dihedral corner, the maximum bouncing time is

(40) $$N_{\max}=\left\lceil \frac{180}{50}\right\rceil =4$$

Considering the equivalent length, the secondary scattering is not the main contribution of the RCS. When the bouncing time reaches 3 or 4, the equivalent length becomes larger. Setting the bouncing time as 3, the angles with high RCS values can be calculated as

(41) $$\begin{array}{l} \theta_{i}+\theta_{s}=\pi -2\beta \cdot 2,for\;plane\;a\\ 2\beta -\theta_{i}+2\beta -\theta_{s}=\pi -2\beta \cdot 2,for\;plane\;b \end{array}$$

For the fourth bouncing time, the angles are calculated as

(42) $$\begin{array}{l} \theta_{s}-\theta_{i}=2\beta \cdot 4-\pi ,for\;plane\;a\\ 2\beta -\theta_{s}-\left(2\beta -\theta_{i}\right)=2\beta \cdot 4-\pi ,for\;plane\;a \end{array}$$

In monostatic case, $\theta_{i}=\theta_{s}$. Using (41), the angles with high RCS values are

(43) $$\begin{array}{l} \theta_{i}=\frac{180-50\times 2}{2}=40{}^{\circ}\\ \theta_{i}=\frac{50\times 4-180}{2}=10{}^{\circ} \end{array}$$

**Figure 16:**
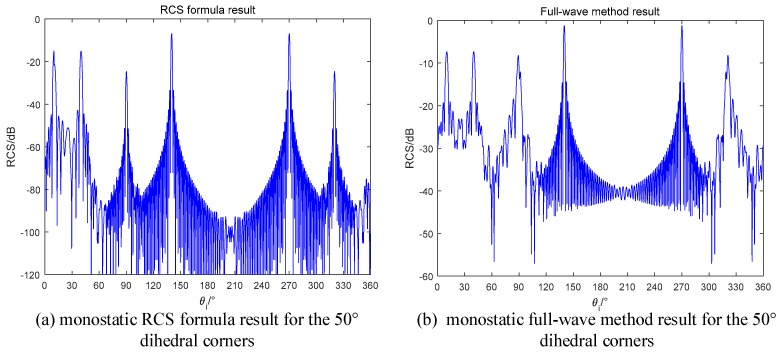
Monostatic RCS of the 50° dihedral (**a**) RCS formula in this paper; (**b**) Full-wave method.

The monostatic RCS simulation results are shown in Figure 15. As can be seen from Figure 16, there are two peaks locate at 10° and 40°. The mismatch around 90° and 320° is caused by the diffraction of electromagnetic wave [26], which is not the focus point in this paper. However, for the RCS values at these points, our formula demonstrates the reduced phenomena by computing the shadow effect. When the incident angle is not in the inner of the dihedrals, two planes will block each other, and the RCS will be reduced.

In bistatic radar case, the fourth reflection can contribute to the RCS when the bistatic angle is designed properly. Based on (42), we have

(44) $$\begin{array}{l} \theta_{s}-\theta_{i}=50\times 4-180=20{}^{\circ}\\ \theta_{i}-\theta_{s}=50\times 4-180=20{}^{\circ} \end{array}$$

**Figure 17:**
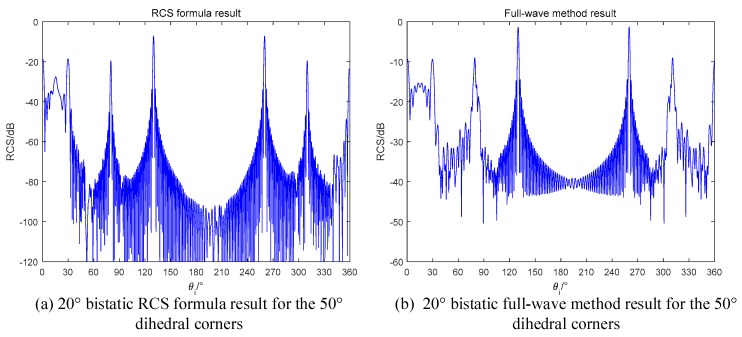
Monostatic RCS of the 50° dihedral (**a**) RCS formula in this paper; (**b**) Full-wave method.

Based on the reciprocal theory of radar scattering, the bistatic angle is assumed as a positive number in this paper. Therefore, if we design a bistatic radar with a 20° bistatic angle, the fourth reflection can be detected in the RCS. The simulation results are shown in Figure 17. In the range of [0, 30°], there is a sinc-like envelope as the same with the curves in Figure 14. Regarding the same behaviors in the even time’s reflection, we have concluded that the bistatic radars have the ability to detect the multiple reflection. In Figure 17, the shadow effect also exists. The RCS value at 80° is smaller than that at 130°. When the incident angle is 80°, the bistatic radar will receive the specular reflection of plane a. However, plane b blocks some of the illumination and a shadow on plane a occurs. Thus, the specular reflection of plane a is no longer as the full-illuminated condition, and the RCS is reduced.

### 3.3. Summary

In this section, we have established the analytical RCS formula for the dihedral corners. Starting from a single plane to the multiple bouncing effect, we have analyzed the contribution of each plane and equivalent plane for the final RCS and used the mathematical formulas to make the computation of RCS in an explicit expression.

From orthogonal to acute dihedral, we have compared our formula with the full-wave method and the results show that our theory is in good agreement with the real physic.

Next, it is necessary to analyze the representation of the dihedral corner reflectors in SAR images.

## 4. SAR Image Simulation of Dihedral Corners

In this section, we analyze the SAR imaging results of orthogonal, obtuse and acute dihedral corners, and compare their similarities and differences of SAR images under the monostatic and bistatic conditions. The echoes data is obtained by the Method of Moment (MoM) [25]. The focusing of the echoes data is processed by BP (Back-Projection) algorithm, which is a robust SAR focusing method. Even it is not very fast, the BP algorithm is suitable for many extreme geometric configurations, especially for bistatic SAR sensors [27]. The BP algorithm can be divided into two processing steps: range compression and azimuth projection. The range compression is the matched filtering applying in the range direction while the azimuth projection is to make the range compression results projection into the image domain according the positions of the transmitters and receivers. The details can be referred to [27].

**Figure 18:**
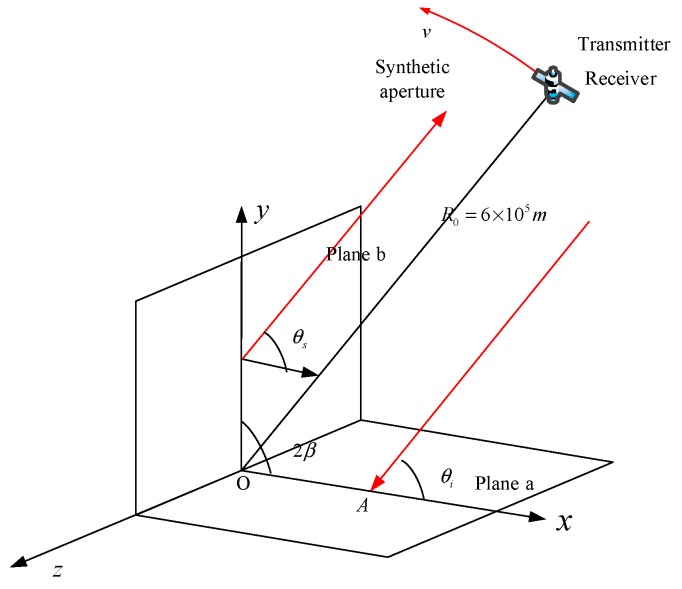
Sketch map of the SAR imaging simulation.

As for the geometric configuration of our simulations, it is shown in Figure 18. In order to cover most situations, the transmitter and the receiver are depicted as a separated pair with a bistatic angle. In the monostatic mode, they are in the same position and fly in the same velocity. In the bistatic mode, the receiver and the transmitter are flying in the pursuit mode, where they fly with the same velocity along the circular track and with a fixed bistatic angle. The dihedral targets are located at the original point of the object coordinate. In simulation experiments, we fix the size of the dihedral corner, whose plane length is 20λ = 0.6 m, and change the opening angles.

The simulate radar system is set as a circular SAR. The parameters of the circular radar are shown in Table 2. To make the simulation match the practical situation, we use the system parameters for a X-band spaceborne radar system. Instead of using velocity to describe the movement of the SAR, we use the angle interval and the aperture range, which are determined by the velocity, the synthetic aperture time and the pulse repetition frequency. The details can be referred to [23].

### 4.1. Orthogonal Dihedral Corner

**Figure 19:**
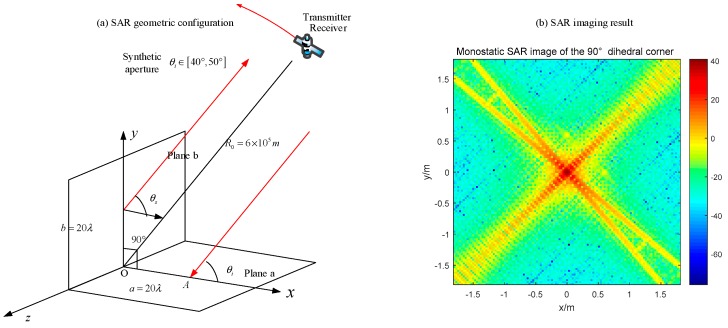
Monostatic image of the orthogonal dihedral corner (**a**) geometric configuration; (**b**) imaging result.

In the first simulation, we set the middle object as an orthogonal dihedral corner, and the synthetic aperture is from 40° to 50°. Based on the wave path analysis in Section 2, it is predicted that the SAR image under this condition will be a strong point with the radar system response, which is called as PSF (Point Spread Function). This is the typical characteristic of dihedral corners in SAR images, and we call it the dihedral effect. The dihedral effect is a phenomenon that when a SAR senor illuminate a dihedral target, its SAR image acts as a point target with strong pixel value, and all the geometry information about the dihedral target is invisible in SAR images (Figure 19).

**Figure 20:**
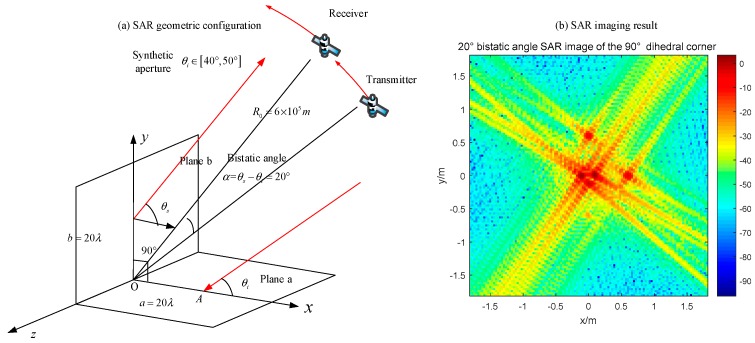
20° bistatic angle SAR image of the orthogonal dihedral corner (**a**) geometric configuration; (**b**) imaging result.

All the images are displayed in dB. In Figure 19, we can just see a point with its SAR PRF, and the value of the original point is around 40 dB. As for the bistatic radar with a 20° bistatic angle (Figure 20), the SAR image has several obvious points which correspond to the endpoints of two panels. Moreover, the maximum pixel value is much lower than that in Figure 19.

The monostatic SAR image of the orthogonal dihedral generates a strong point-like target, which is quite different with its geometric shape. The point-like image coincides with the previous analysis, which indicates that the brightest point locates at the connection point of the dihedral corner. For the bistatic SAR, the pixel values are much lower than the monostatic one and the image responses to the endpoints of two planes. Therefore, it can be concluded that monostatic SAR sensors are more sensible for orthogonal dihedral corners.

### 4.2. Obtuse Dihedral Reflector

**Figure 21:**
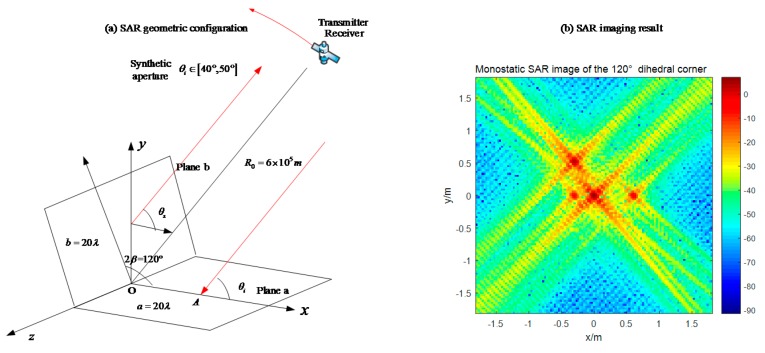
Monostatic SAR image of a 120° dihedral corner (**a**) geometric configuration; (**b**) imaging result.

**Figure 22:**
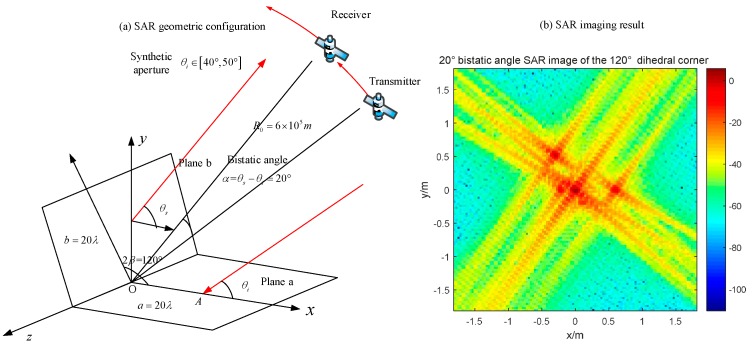
20° bistatic angle SAR image of the 120° obtuse dihedral corner (**a**) geometric configuration; (**b**) imaging result.

**Figure 23:**
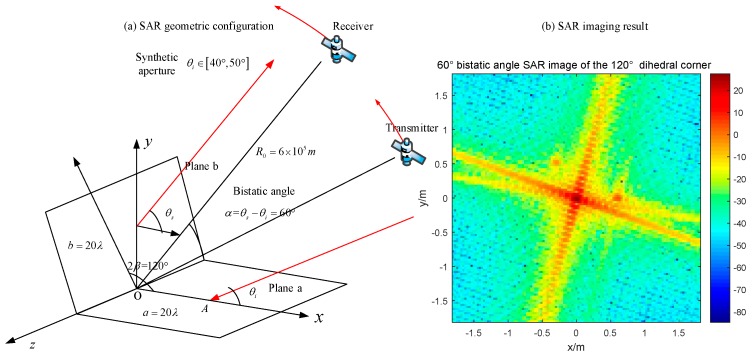
Bistatic SAR with 60° bistatic angle image of the 120° obtuse dihedral angle (**a**) geometric configuration; (**b**) imaging result.

Changing the opening angle of the reflector, its behavior in SAR images becomes different. Setting the experimental object as an obtuse dihedral corner reflector, we use monostatic SAR and bistatic SAR to image it. Based on the previous analysis, the monostatic SAR for this target will not appear the dihedral effect, such as in Figure 19. In Figure 21, the maximum pixel value is around 0 dB. If the bistatic angle is not suitable for this dihedral corner, there will be the same situation. At first, we use an unsuitable configuration, 20° bistatic angle, to image it. The result is shown in Figure 22. The pixel value of the original point in Figure 22 is also around 0 dB, which is almost no difference with the monostatic case. It is difficult to get the dihedral effect under inappropriate bistatic angles. According to the conclusions in Section 3.2.2, the proper bistatic angle should be 60°. The SAR images under the 60 ° bistatic angle configuration are shown in Figure 23.

In Figure 23, the appropriate bistatic angle is used and the obtuse dihedral angle is presented as a strong point, which means the dihedral effect appears. Furthermore, only the bistatic SAR with specific bistatic angle can get the dihedral effect and its bistatic angle is depending on the opening angle. The relationship has been discussed in Section 2.4.

### 4.3. Acute Dihedral Reflector

**Figure 24:**
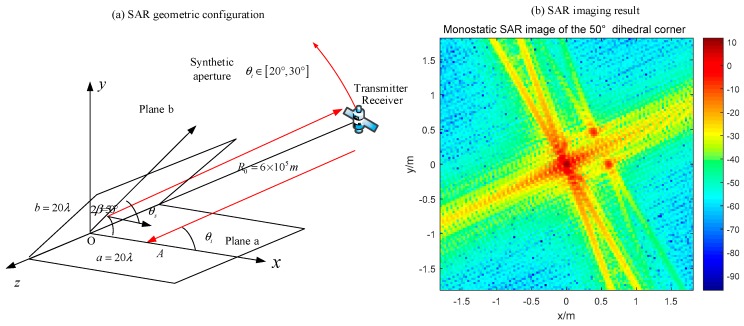
Monostatic SAR image of a 50° dihedral corner (**a**) geometric configuration; (**b**) imaging result.

As for the acute dihedral corner reflectors, the multiple reflection occurs. Based on the discussion in Section 2.3, the radar can receive high RCS only when the bistatic angle is properly designed. In that case, the dihedral effect will appear in the SAR image. For the acute structure discussed in Section 3.2.3, we use the same radar configurations to image the target. The simulation of the monostatic SAR is shown in Figure 24.

**Figure 25:**
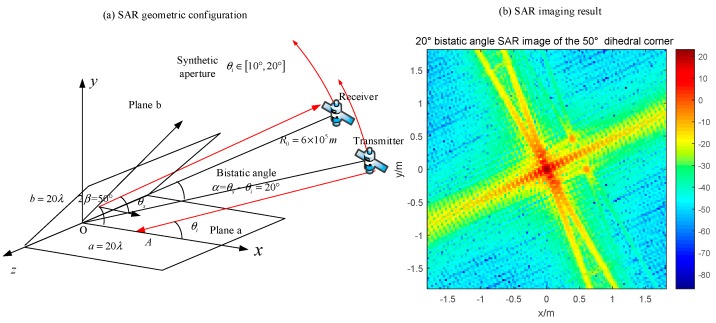
20° bistatic angle SAR image of a 50° dihedral angle (**a**) geometric configuration; (**b**) imaging result.

The monostatic radar moves from 20° to 30° where it cannot receive the third reflection. Therefore, the pixel value of the original point is lower than the dihedral effect which occurs in Figure 19 and Figure 23. According to the discussion in Section 3.2.3, the dihedral effect of this acute dihedral corner can be obtained by using the bistatic SAR with a 20° bistatic angle. The results are shown in Figure 25. The transmitter moves from 10° to 20°, which makes the fourth reflection caught by the bistatic radar. The dihedral effect is obvious in Figure 24 where the highest value is around 20 dB, while the case of monostatic SAR is 10 dB.

### 4.4. Real Data Validation

**Figure 26:**
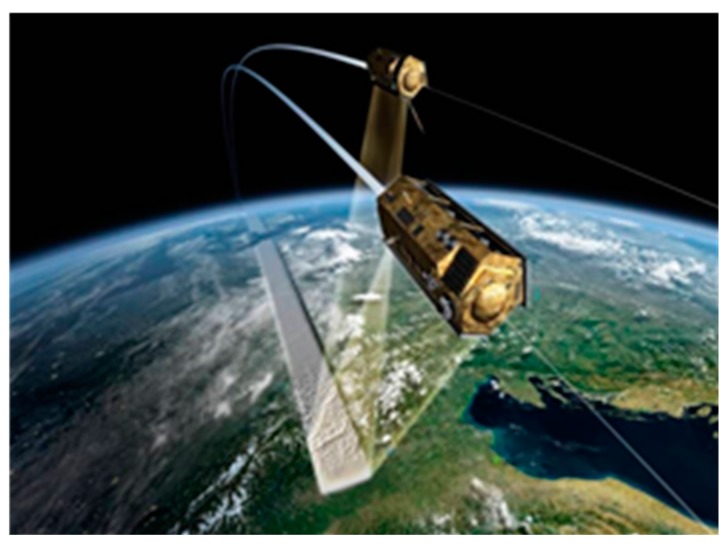
Configuration of TanDEM-X bistatic system (www.dlr.de).

In this subsection, we have applied TanDEM-X data to test our theory. The TanDEM-X is an on-orbit satellite system developed by Germany Aerospace Centre (DLR) and it had been launched in 2010 [28]. Especially, the TanDEM-X has two satellites and has the ability of bistatic acquisition. The geometric configuration of TanDEM-X is shown in Figure 26. TanDEM-X system has two satellites, a master and a slaver. The master satellite, which is TerraSAR-X satellite, sends radar waves and receives the echoes while the slave satellite only receives the echoes. Therefore, TanDEM-X data has two modes: monostatic mode and bistatic mode.

**Figure 27:**
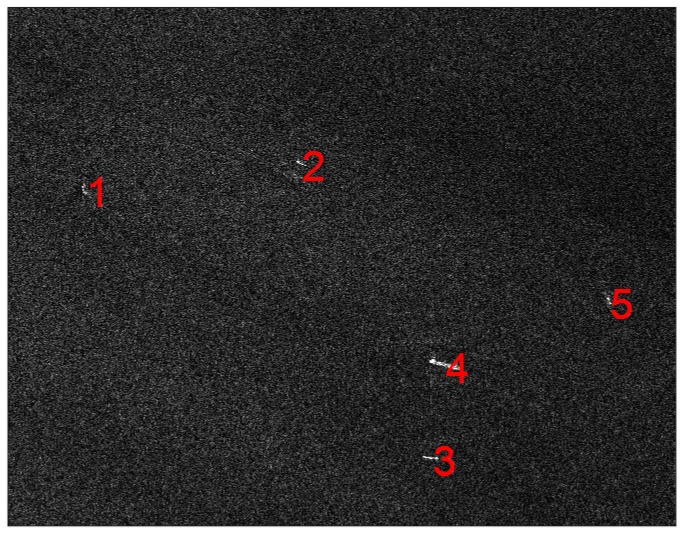
The five labeled ship targets in TanDEM-X image.

With this bistatic radar system configuration, we can use TanDEM-X data to investigate the RCS difference between monostatic mode and bistatic mode. The baseline of two satellites is 300 m, and the initial distance from the satellite to the target is about 610 km. Thus, the bistatic angle of the TanDEM-X product is

(45) $$\alpha =\frac{baseline}{R_{0}}=0.08{}^{\circ}$$

where $baseline=300\;m,R_{0}\;=\;6.1\;\times \;10^{5}\;m$. Ships in the sea are chosen as examples to show the difference of their pixel values under the two modes, considering the ships contain many dihedral structures and their surrounding environment is the sea which is in low RCS and would not influence the pixel value of the ships. The ships in the TanDEM-X SAR image are shown and labelled in Figure 27.

**Figure 28:**
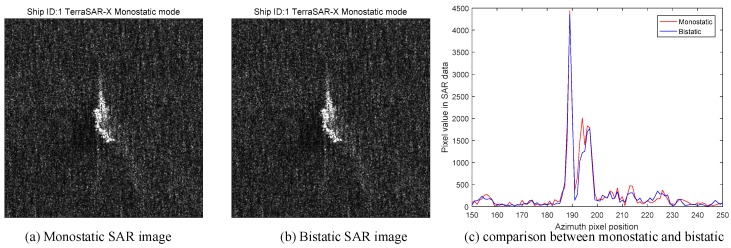
Comparison results of ship 1 (**a**) monostatic; (**b**) bistatic; (**c**) comparison curves.

For each ship, we compare its pixel value, which responses to the RCS under the corresponding SAR acquisition. If the pixel value is higher, it means the RCS in the pixel is larger. The comparison of ship 1 is shown in Figure 28.

**Figure 29:**
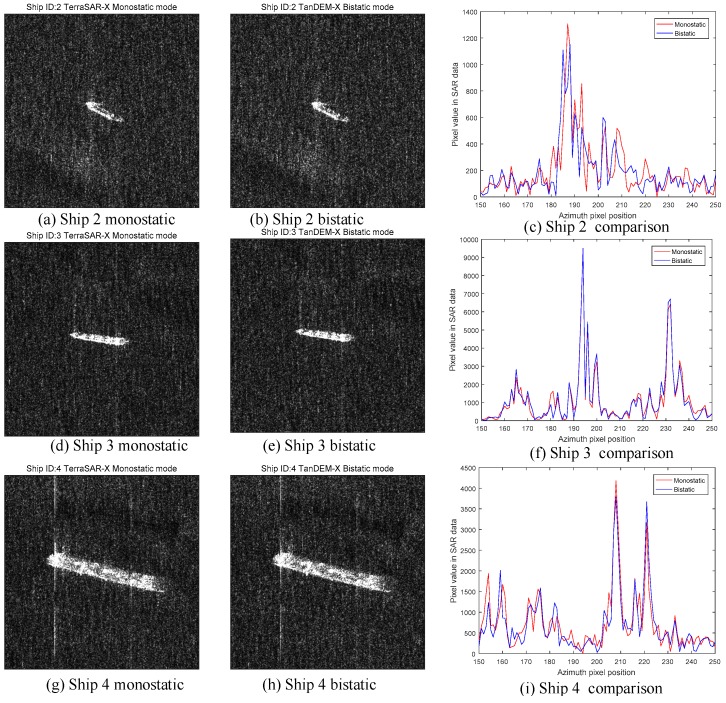

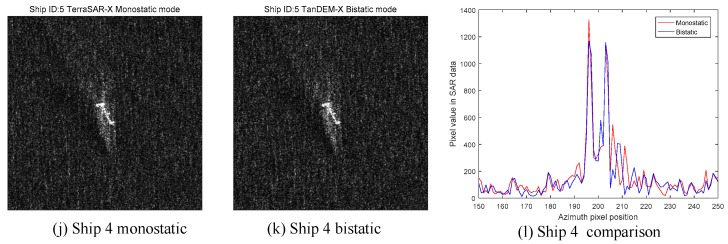
Comparison results of ship 2, 3, 4 and 5 (**a**) Ship 2 monostatic; (**b**) Ship 2 bistatic; (**c**) Ship 2 comparison curves; (**d**) Ship 3 monostatic; (**e**) Ship 3 bistatic; (**f**) Ship 3 comparison curves; (**g**) Ship 4 monostatic; (**h**) Ship 4 bistatic; (**i**) Ship 4 comparison curves; (**j**) Ship 5 monostatic; (**k**) Ship 5 bistatic; (**l**) Ship 5 comparison curves.

In Figure 28, we can see that the monostatic SAR image and the bistatic SAR image of ship 1 are almost the same. However, Figure 28c tells the difference between two images. Figure 28c has two curves, which is the pixel value sliced from the central point along the azimuth direction. As for the peaks of two curves, the peak of the monostatic SAR image has larger pixel value than the bistatic image. For a cargo ship, the most common structures are dihedral corners generated by the plane, the deck and the cabin. Usually, they are in right-angle geometry. According to the theory in this paper, the monostatic SAR has a higher RCS response for the orthogonal dihedral corner than the bistatic one. Therefore, the pixel value curve of the monostatic SAR is higher than that of bistatic SAR in Figure 28c, which fits our theory well. In order to make it more credible, we use more ships to validate our prediction. The other ships are shown in Figure 29. They all have the similar performances. Overall, according to the results in Figure 28 and Figure 29, we can conclude that the bistatic SAR will generate lower amplitude SAR image than the monostatic SAR for the right-angle dihedral corner structures.

## 5. Discussion

Dihedral corner is the fundamental structure of many targets. It has important value of studying its scatting characteristics, especially for the bistatic SAR systems. Through theoretical analysis and experimental validation, we have investigated three kinds of dihedral corners. They are classified by the opening angle: orthogonal, obtuse and acute.

As for the orthogonal dihedral corners, they are the most common ones whose opening angle is 90°. According to the principle of microwave propagation, the wave reflects along the incident direction inversely in this structure. Therefore, the monostatic radar systems can easily detect this kind of targets. In the monostatic SAR images, the orthogonal dihedral corner performs as a point target with high RCS, which is called the dihedral effect in this paper. In the bistatic SAR images, the detected RCS is low and the endpoints of the reflector are displayed. In order to verify our theory, TanDEM-X data is occupied. The monostatic and bistatic modes of TanDEM-X data show that the dihedral corners in ships, usually the orthogonal dihedral corners, have higher RCS in the monostatic images than the bistatic images.

As for the obtuse dihedral corners, they are often designed for stealth targets. Acting as a method to reduce RCS, the RCSs of obtuse dihedral corners are quite low when observed by the monostatic SAR. However, when using a bistatic SAR, the hidden RCS could be detected, and obtuse dihedral corners will show the dihedral effects in the SAR images. It is important that the bistatic angle should be properly designed based on the opening angle. Because the double bounce is the maximum reflection in the obtuse dihedral corners, the design for bistatic SAR is α = 4β−π, where α is the bistatic angle.

As for the acute dihedral corner, it is quite different with the other twos. The multiple reflections larger than two times arise in this structure, which make the propagation of the radar wave complicate. In this paper, we extend the RCS calculation formula for the multiple reflection in the acute dihedral corners and give the formula of general term to depict the relationship between the incident angle and the emergent angle. Based on the accurate analysis of target characteristics, the bistatic SAR can be designed in the following formula,

(46) $$\left\{ \begin{array}{l} \theta_{s}+\theta_{i}=\pi -2\beta \left(n-1\right),n=2k-1\\ \theta_{s}-\theta_{i}=2\beta n-\pi ,n=2k \end{array}\right.,k\in N^{+}$$

The design of bistatic radar depends on the scattering times. The orthogonal and the obtuse dihedral corners are the special cases of the acute dihedrals, where only double reflection occurs. Guiding by the formula, we select the proper bistatic angle to image an acute dihedral corner and the dihedral effect of the SAR image is obtained in the simulation.

Based on the maximum pixel value of the SAR image with the dihedral effect, the orthogonal dihedral corner is the highest, which reaches 40 dB (Figure 19). The values of the acute dihedral corner and the obtuse dihedral corner are around 20 dB (Figure 23 and Figure 25). It is because that the designed bistatic radar catches the main echo responding for one single plane while the monostatic radar receives the main echo responding to both planes of the orthogonal dihedral. Besides, all the three targets have the same plate size and the only difference is the opening angle. Therefore, it is concluded that the dihedral effect of SAR images is related to the opening angle. These are preliminary findings, and more research is needed.

## 6. Conclusions

In this paper, we analyse the radar scattering characteristics of the dihedral corner reflectors in bistatic SAR images, and qualitatively give the scattering mechanism of dihedral corners. A quantitative RCS formula is used to compare the SAR image features of the dihedrals with different opening angles.

Changing the angle of the dihedral is a very effective stealthy approach that changes the direction of the echo and causes the monostatic radar to lose its ability to detect such structures. Because of the flexible geometric configuration, bistatic radars have the ability to catch the main energy of a un-rectangle dihedral corner. In this paper, based on the principle of mirror scattering in geometrical optics, the propagation path of radar beam in dihedral corners is analysed, and the general formula of bouncing time and final emergent angle is given, which has the relationship with the opening angle and the incident angle. Secondly, based on the idea of equivalent plane, the formulas of RCS for bistatic radars with different angles are given quantitatively. The correctness of the formula are verified by comparing with the full-wave method. Finally, the echo data of different dihedral corners are simulated, and SAR imaging is carried out. In a particular radar geometry, the dihedral angle image can be expressed as a strong point at the corner, and these specific angles are consistent with the obtained through the analysis of the geometrical optics. At the same time, the TanDEM-X SAR data is used for testing our theory.

Based on the accurate analysis of the target scattering characteristics of dihedral corners in bistatic configuration, the RCS and the corresponding methods of how to a design bistatic radar are given. The anti-stealth performance of bistatic radar is theoretically demonstrated. However, this paper only considers dihedral structures, and there is no discussion about other targets. The future work is to analyze the relationship between the maximum value in dihedral effect and the opening angle, and the bistatic scattering characteristics of other typical structures, then make full use of the flexible configuration of bistatic radar to detect and identify targets better.

## Figures and Tables

**Table 1:** Explanation of the parameters in Formula (29).

*M*	*S*	$R_{m}$	$P_{m}$ (Horizontal)	$Q_{m}$
1	$S_{a}$	ka	$\theta_{i}$	$ka\left[\cos\theta_{i}+\cos\theta_{s}\right]/2$
2	$S_{b}$	kb	$2\beta -\theta_{i}$	$kb\left[\cos\left(2\beta -\theta_{i}\right)+\cos\left(2\beta -\theta_{s}\right)\right]/2$
3	$S_{ab}$	$kb^{\prime }$	$2\beta +\theta_{i}$	$kb^{\prime }\left[\cos\left(2\beta +\theta_{i}\right)+\cos\left(2\beta -\theta_{s}\right)\right]/2$
4	$S_{ba}$	$ka^{\prime }$	$4\beta -\theta_{i}$	$ka^{\prime }\left[\cos\left(4\beta -\theta_{i}\right)+\cos\theta_{s}\right]/2$

**Table 2:** System parameters of the SAR simulation.

Carrier frequency	9.65 GHz
Bandwidth	2 GHz
Frequency stepping interval	40 MHz
Azimuth angle range	0~359°
Angle interval	0.1°
Initial range distance	600 km
